# Implementing Precision Regional Anesthesia in an Emergency Setting: Bilateral Upper Trunk Blocks for Shoulder Reduction

**DOI:** 10.7759/cureus.97859

**Published:** 2025-11-26

**Authors:** Kartik Sonawane

**Affiliations:** 1 Anesthesiology, Ganga Medical Centre and Hospitals Pvt. Ltd., Coimbatore, IND

**Keywords:** bilateral shoulder dislocation, phrenic nerve-sparing, regional anesthesia in emergency care, ultrasound-guided nerve block, upper trunk block

## Abstract

Bilateral anterior shoulder dislocations (BASDs) are rare orthopedic emergencies that pose significant anesthetic challenges, particularly when standard sedation or general anesthesia (GA) is contraindicated due to non-fasted status or airway risk. A young, healthy male presented to the emergency department with radiologically confirmed bilateral anterior glenohumeral dislocations following a fall. He reported severe bilateral shoulder pain (visual analogue scale (VAS) 7/10 at rest, 10/10 on movement) but was cooperative, hemodynamically stable, and nil per os (NPO). Given the risk of aspiration, regional anesthesia (RA) was selected. Ultrasound-guided bilateral upper trunk blocks (UTBs) were administered using 3 mL of 2% lidocaine per side, without sedation, opioids, or premedication. Complete analgesia was achieved within minutes, preserving motor function and airway reflexes. Pain-free bilateral shoulder reductions were performed successfully. The patient was discharged 10 minutes post-procedure following two hours of observation. No adverse effects, including phrenic nerve palsy, were observed. While interscalene blocks remain the standard for shoulder analgesia, they are contraindicated bilaterally due to the near-universal risk of diaphragmatic paralysis. UTB, targeting the C5-C6 roots, provides selective analgesia to the suprascapular and axillary nerves with minimal risk to the phrenic nerve. This case illustrates the safety, efficacy, and practicality of bilateral UTB as a phrenic-sparing, opioid-free alternative in select BASD cases. In appropriately selected patients, bilateral UTB may offer a safe, airway-conscious alternative to GA or sedation for shoulder reduction, particularly in non-NPO or resource-constrained environments. This technique warrants further validation as a frontline option in emergency RA practice.

## Introduction

Shoulder dislocations are among the most frequent orthopedic emergencies encountered, accounting for nearly 50% of all large joint dislocations in clinical practice [1]. However, bilateral anterior shoulder dislocations (BASDs) are exceptionally rare, with fewer than 150 cases reported in the literature [2,3]. This rarity stands in sharp contrast to unilateral anterior dislocations, which comprise over 95% of glenohumeral dislocations [1]. BASDs typically result from simultaneous, symmetrical trauma, seizures, or electric shocks [4,5]. The largest available review involving 87 cases documented a mean age of 41 years, with a male predominance (72%) and seizure-related causes accounting for 31% of cases, whereas trauma-induced BASD was more common in elderly females [2,5]. The acute pain, functional limitation, and urgency of reduction demand swift, effective, and safe analgesia.

The orthopedic reduction of shoulder dislocations, although straightforward, is complicated by anesthetic challenges, particularly in bilateral presentations. Patients often arrive in the emergency department (ED) non-fasted, distressed, and with varying levels of cooperation or comorbidities. Conventionally, procedural sedation or general anesthesia (GA) is employed to facilitate shoulder relocation, especially in bilateral cases where cooperative positioning is limited [6,7]. However, these approaches are not without risk - particularly when the patient presents inadequately fasted or harbors comorbid conditions that raise concern for airway or hemodynamic compromise [8,9].

While various RA techniques exist for shoulder procedures - such as interscalene block (ISB) [10], selective suprascapular block [11], combined axillary-suprascapular block [12], and shoulder pericapsular nerve group (PENG) block [13] - most are suboptimal in emergency bilateral reductions due to either high risk of phrenic nerve involvement or incomplete nerve coverage. Other approaches often provide incomplete anterior capsule coverage or require high-volume injections near critical structures. The upper trunk block (UTB), targeting the proximal upper trunk, offers selective analgesia with minimal respiratory compromise [14,15], positioning it as a promising alternative for bilateral dislocations, especially when GA or sedation is not feasible [16].

This case report underscores a real-time clinical pivot in response to such a scenario: a young, healthy adult presenting with simultaneous BASDs but not adequately nil per os (NPO). Faced with the challenge of avoiding sedation-related complications while ensuring patient comfort and procedural success, a bilateral UTB was employed as the sole anesthetic technique - a decision grounded in precision regional anesthesia (RA) and phrenic-sparing strategy [17,18]. What followed was a fast, effective, motor-sparing, and sedation-free shoulder reduction, completed within minutes of the block, without compromising airway safety or prolonging ED stay. This report highlights the expanding role of RA in emergency care, especially in non-operating room settings, and proposes UTB as a viable, safe, and efficient alternative for BASDs in carefully selected patients.

## Case presentation

A young (in his 30s) healthy male presented to the ED with complaints of bilateral shoulder pain and inability to lift both arms following a slip and fall from steps at home. The incident occurred in the afternoon while he was descending the stairs barefoot, resulting in a forceful impact on both outstretched arms. On arrival, he held both upper limbs in adduction and internal rotation, with severe pain and visible anterior deformities. He was conscious, oriented, hemodynamically stable, and cooperative.

**Figure 1:**
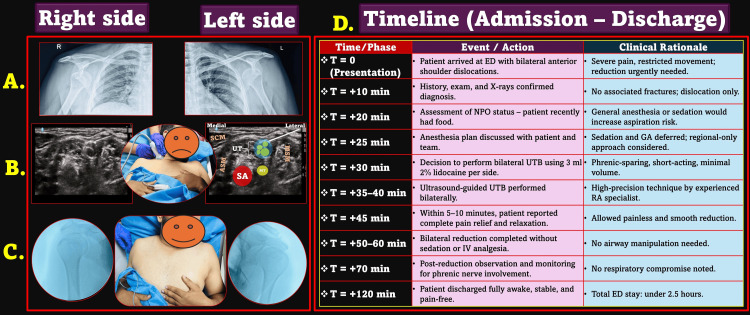
Clinical Timeline and Procedure of the Bilateral Upper Trunk Block (UTB). A. X-rays showing bilateral anterior shoulder dislocations (right and left). B. Ultrasound-guided upper trunk block (UTB) performed bilaterally: Left side schematic shows sonoanatomy with probe placement. C. Post-reduction image showing successful reduction on both sides and a comfortable, pain-free patient. D. Timeline summarizing admission, block administration, reduction, monitoring, and discharge with corresponding clinical rationale. SCM, sternocleidomastoid; ASM, anterior scalene muscle; MSM, middle scalene muscle; UT, upper trunk; SA, subclavian artery; MT, middle trunk; ED, emergency department; NPO, nil per oral; RA, regional anesthesia; GA, general anesthesia; USG, ultrasound guidance Source: This figure was created by the author, KS.

Initial clinical assessment confirmed BASDs, which were subsequently verified with radiographs (Figure 1A). According to the visual analogue scale (VAS), his pain was rated 7/10 at rest and increased to 10/10 with any attempted movement. He denied any loss of consciousness, vomiting, ENT bleeding, or seizures. Notably, he had a prior history of right shoulder dislocation five years ago, which had self-resolved, and no history of sleep dislocations or comorbid illnesses. His NPO status was not adequate at the time of arrival, raising concerns about aspiration risk with sedation or GA.

In light of this, a RA-only approach was adopted. After explaining the plan to the patient and obtaining informed consent, bilateral ultrasound-guided UTBs were performed using a lateral-to-medial in-plane approach (Figure 1B). A total of 3 mL of 2% lidocaine was administered per side at the level of the upper trunk (C5-C6), carefully avoiding spread toward the anterior scalene muscle and phrenic nerve. Written informed consent was obtained from the patient for publication of this anonymized case and associated clinical images.

Within minutes, the patient experienced complete pain relief (VAS 0/10) without motor weakness. The orthopedic team performed closed reductions smoothly and painlessly under RA alone, without requiring sedation, systemic analgesia, or airway intervention. The patient expressed surprise that the procedure had already concluded (Figure 1C). The patient stood and walked without distress and was discharged from the ED, just 10 minutes after the reduction.

Post reduction, he was observed for signs of phrenic nerve involvement or other complications. No respiratory compromise occurred, and the patient remained stable, fully awake, and pain-free. He was discharged from the ED the same evening with arm slings and follow-up advice. The entire clinical sequence, including presentation, block performance, procedural success, and discharge, is illustrated in Figure 1D.

## Discussion

This unique case underscores the dynamic role of RA in emergency orthopedic settings, especially in scenarios where conventional sedation or GA may be suboptimal or even hazardous. The successful execution of bilateral ultrasound-guided UTBs for managing simultaneous anterior shoulder dislocations in a conscious, cooperative patient not only challenges traditional paradigms but also represents a significant advancement in patient-centric, resource-efficient acute care. Importantly, it illustrates how anatomical precision can circumvent the need for systemic agents, supporting a strategic move toward phrenic nerve-sparing techniques in bilateral upper limb procedures.

Historically, bilateral above-clavicle blocks have been discouraged due to the high incidence of phrenic nerve palsy - nearly 100% even with low-volume ISBs [19,20]. UTBs, however, target the upper trunk (C5-C6) away from the anterior scalene, resulting in a significantly lower incidence (~10-20%) of diaphragmatic involvement [14,18,21,22]. In this case, precise deposition of just 3 mL of 2% lidocaine per side achieved effective analgesia by targeting the axillary and suprascapular nerves (C5-C6) - key contributors to shoulder joint innervation [23,24] - while sparing the musculocutaneous nerve (C5-C7) and thus preserving motor function. Lidocaine’s rapid onset and short duration made it an ideal agent for a brief, conscious procedure without lingering motor or respiratory compromise.

Pain during shoulder dislocations arises from both capsular injury and muscular spasm [25], primarily mediated by nerves such as the axillary, suprascapular, subscapular, lateral pectoral, and musculocutaneous branches [23,24]. Without appropriate management, these pain sources can resist reduction and increase the risk of secondary injury. Muscle groups such as the deltoid, subscapularis, pectoralis major, and short head of the biceps often contract reflexively, creating substantial resistance to realignment [25-27].

**Figure 2:**
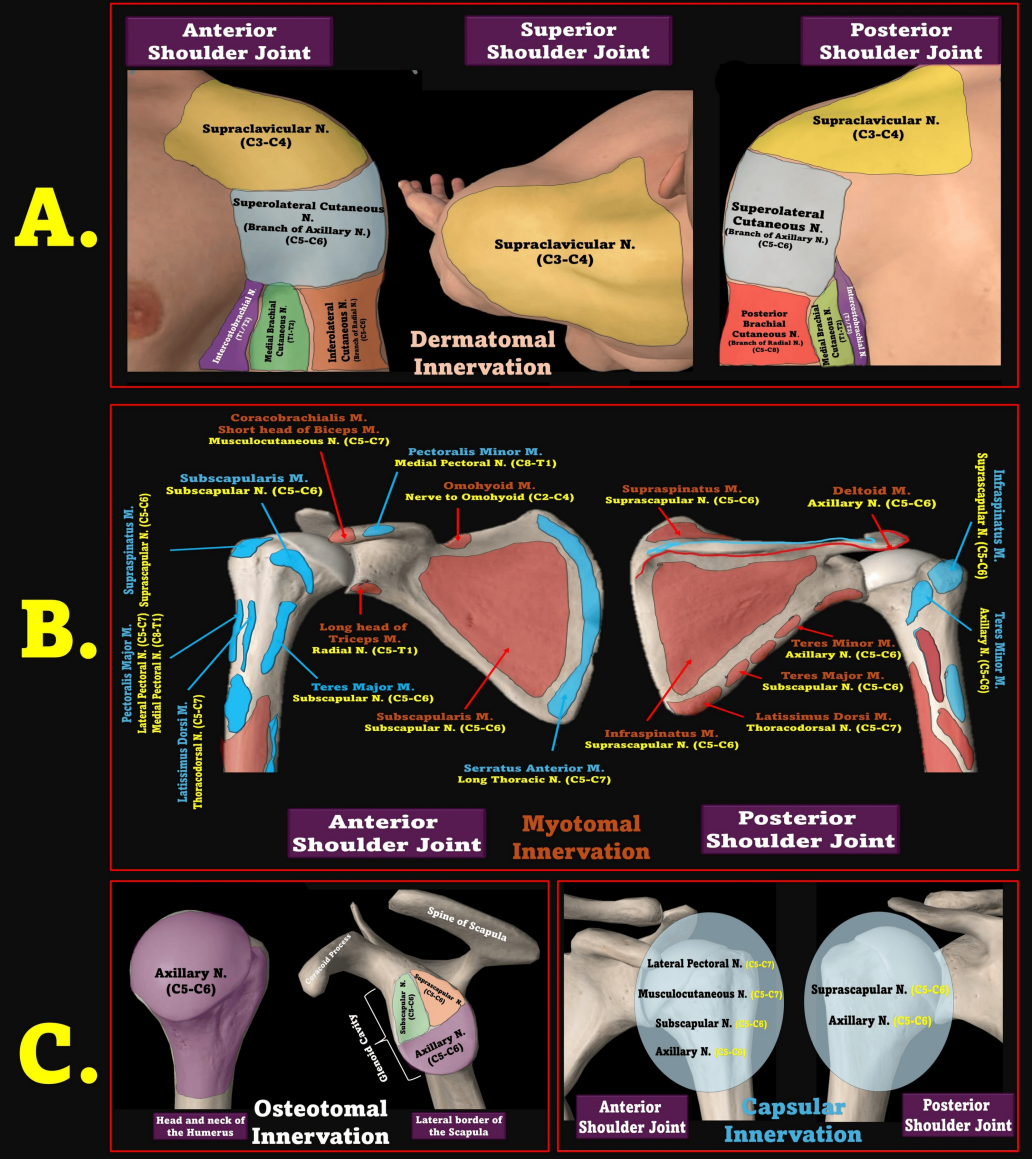
Multilayered Innervation of the Shoulder Joint. A. Dermatome map showing cutaneous innervation of the anterior, superior, and posterior shoulder regions. B. Myotomal innervation of muscles around the shoulder joint, including contributions from C5–T1 spinal roots. C. Osteotomal and capsular innervation patterns. N, nerve; M, muscle; SCM, sternocleidomastoid; UT, upper trunk; RA, regional anesthesia Source: This figure was created by the author, KS.

Traditionally, pharmacologic strategies, including procedural sedation (opioids/benzodiazepines) or GA, have been employed to overcome this resistance. In seizure-induced BASDs, intravenous agents such as ketamine or propofol have achieved high success rates (~80%) for closed reductions [3,28]. However, in non-fasted patients, these systemic strategies increase aspiration risk and necessitate post-procedure monitoring. In contrast, the UTB enabled complete analgesia, muscle relaxation, and patient cooperation - without compromising airway safety or requiring systemic medication. Figure 2 illustrates the multi-level innervation involved in shoulder pain, reinforcing the selective advantage of UTB.

**Figure 3:**
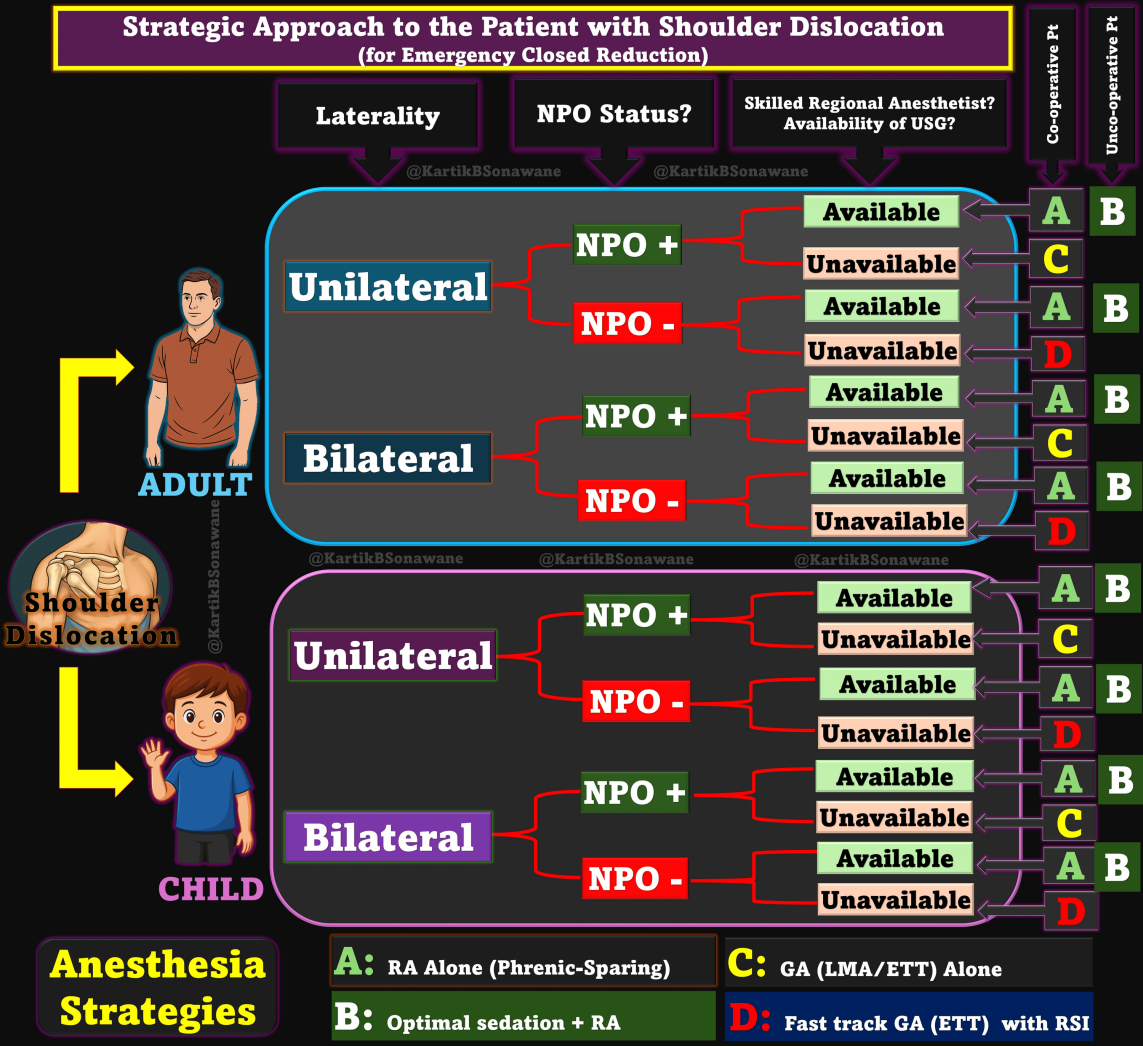
Strategic Approach to Shoulder Dislocation Management Based on Laterality and NPO Status. A clinical decision matrix for adults and children presenting with shoulder dislocations, integrating laterality (unilateral vs. bilateral), NPO status, availability of skilled regional anesthetist, and patient cooperation to guide the optimal anesthesia strategy—ranging from phrenic-sparing regional techniques to general anesthesia. NPO, nil per oral; GA, general anesthesia; RA, regional anesthesia; RSI, rapid sequence intubation; ETT, endotracheal tube; LMA, laryngeal mask airway; USG, ultrasound guidance Source: This figure was created by the author, KS.

While GA remains a viable option - especially in uncooperative or complex cases - it comes with logistical demands, including fasting, airway instrumentation, and postoperative recovery, which often extend the ED stay. Sedation similarly delays discharge and introduces risks when fasting status is uncertain. Conversely, RA using UTB provided a conscious, opioid-free alternative that allowed immediate assessment and recovery. Even in delayed or seizure-related dislocations, RA can serve as a bridge to definitive management, particularly in high-risk patients. Figure 3 presents a proposed decision-making algorithm specifically designed for such scenarios.

Multiple RA techniques have been described for shoulder procedures; however, each has limitations in bilateral or emergency applications. The ISB is unsuitable for bilateral use due to its high risk of phrenic palsy [10]. Suprascapular and shoulder PENG, although promising, offer limited anterior capsule coverage and require more experience to perform in emergency situations [11,13]. Supraclavicular blocks provide broader analgesia but again risk phrenic nerve involvement [29,30]. Intra-articular injections may relieve pain but do not address muscle spasm, a key barrier to successful reduction [31,32]. Moreover, combining blocks or performing extensive RA in uncooperative or non-NPO patients raises safety and efficiency concerns. UTB, with its focused coverage and minimal volume, mitigates these issues while preserving spontaneous breathing and motor function - an ideal balance in the ED setting.

A pivotal consideration in this case was the patient’s incomplete NPO status - a common issue in EDs, where fasting protocols are often impractical. While GA under rapid sequence induction remains an option, it is invasive and resource-intensive and may unnecessarily extend patient stay. Gastric ultrasound could potentially serve as a point-of-care adjunct to assess stomach contents and refine decision-making; however, in its absence or when equivocal, non-sedative options become critical. Here, RA preserved airway reflexes, consciousness, and cardiorespiratory stability. The use of short-acting LAs further enhanced safety by limiting the potential impact of any inadvertent phrenic nerve involvement. The result was a swift, opioid-free reduction of bilateral dislocations without sedation or compromise to patient safety.

Several factors contributed to the decision to proceed with bilateral UTBs: a cooperative patient, a short and noninvasive procedure, the absence of respiratory comorbidities, and the presence of an experienced RA practitioner. With over a decade of clinical proficiency, the operator minimized risks such as LA spread to the phrenic nerve or intravascular injection. Even in the unlikely event of diaphragmatic paresis, the patient’s healthy reserve and the short duration of lidocaine’s action would allow conservative management. If needed, noninvasive ventilation or ultrasound-guided washout near the phrenic nerve could serve as potential rescue options [33,34].

With growing literature supporting low-volume, ultrasound-guided regional techniques for phrenic-sparing shoulder analgesia [18], UTB emerges as a promising tool in scenarios involving acute bilateral dislocation. This case advocates a nuanced, anatomy-driven approach to emergency shoulder dislocations, particularly those involving bilateral injuries. Rather than defaulting to GA or systemic sedation, clinicians equipped with RA expertise and ultrasound guidance can consider UTB as a first-line option in cooperative, non-NPO patients. Emerging techniques such as the anterior suprascapular block or shoulder PENG block warrant further exploration in bilateral contexts. However, the current case sets the stage for comparative studies validating UTB’s safety, efficacy, and reproducibility in broader clinical scenarios.

The key strength of this report is the demonstration of a safe, phrenic-sparing bilateral regional technique that enables conscious reduction and early discharge without the need for systemic analgesics. The use of a short-acting LA highlights its suitability for quick-turnaround emergency procedures. While formal diaphragmatic imaging was not performed, the patient was closely monitored for respiratory symptoms, and no clinical signs of phrenic nerve involvement were observed. However, as a single-patient case, these findings may not be generalizable to individuals with comorbidities, anxiety, or limited cooperation. Bilateral above-clavicle blocks also demand high technical expertise and anatomical precision. Larger clinical studies are needed to validate the reproducibility and safety of this approach across varied settings.

## Conclusions

Ultrasound-guided bilateral UTBs offer a practical, safe, and effective alternative to GA or sedation for BASDs, especially when airway safety or fasting status is a concern. By leveraging a low-volume, phrenic-sparing technique with a short-acting LA, we successfully achieved complete analgesia without motor blockade or respiratory compromise. The sedation-free approach enabled immediate post-procedure recovery and early discharge, underscoring the value of RA in emergency orthopedic care.

While broader clinical validation is warranted, this experience positions UTB as a viable, patient-centered option for managing bilateral dislocations in high-risk or resource-constrained environments. It exemplifies how precision regional techniques can elevate emergency care when efficiency, safety, and individualized planning are paramount.
